# Two-stage laparoscopic resection of giant hepatoblastoma in infants combined with liver partial partition and artery ligation

**DOI:** 10.1186/s12957-021-02156-y

**Published:** 2021-02-25

**Authors:** Yaohao Wu, Lexiang Zeng, Ronglin Qiu, Jie Zhang, Jianhang Su, Minyi Liao, Xiaogeng Deng

**Affiliations:** Department of Pediatric Surgery, Sun Yat-Sen Memorial Hospital, Sun Yat-Sen University, Guangzhou, China

**Keywords:** Hepatoblastoma, Laparoscopy, Staged surgery, Infant

## Abstract

**Purpose:**

Laparoscopic resection of giant hepatoblastoma (HB) in children has long been a subject of controversy. Here, a new procedure of two-stage laparoscopic resection of giant HB in infants was firstly reported and the feasibility was discussed.

**Methods:**

The clinical data of three infants with HB were retrospectively reviewed, all of which received 3–5 cycles of neoadjuvant chemotherapy. Stage 1 laparoscopic selective hepatic artery ligation and liver partial partition were performed. Stage 2 laparoscopic hepatectomy was performed 2 weeks later.

**Results:**

The results demonstrated that (1) the tumors shrank considerably in size and had relatively clear boundaries after neoadjuvant chemotherapy; (2) after stage 1 surgery, the tumor volume further reduced, while the intratumoral necrosis expanded; (3) 2 weeks later, stage 2 laparoscopic hepatectomy was performed successfully; (4) none of the cases had intraoperative complications such as tumor rupture, air embolism, hemorrhage, biliary fistula, or liver failure, and there was no recurrence or metastasis during follow-up.

**Conclusions:**

Two-stage laparoscopic hepatectomy associating selective hepatic artery ligation and liver partial partition for HB in infants has the benefits of small invasiveness, fast recovery, improved safety, and high feasibility. However, more cases and longer follow-up are needed to assess its long-term efficacy.

Hepatoblastoma (HB) is the most common malignant hepatic tumors, accounting for almost 80% of all pediatric liver cancers [1]. Pediatric HB is a type of embryonal neoplasms, and most likely to be diagnosed in the first 5 years of a child’s life [2]. With advances in imaging techniques, neoadjuvant chemotherapy, surgery, and postoperative chemotherapy, the overall survival rate for children with HB has greatly improved. Cisplatin-based chemotherapy and surgical resection provide standard-risk patients with a 5-year overall survival of more than 90% [3]. However, children in the high-risk category, historically, have a dismal overall survival of only 25–40% [4]. Primary hepatic resection is recommended for patients with PRETEXT stages I and II tumors with no additional annotative risk factors [5]. Otherwise, patients should undergo neoadjuvant chemotherapy and delayed surgery [5]. Lung is a common site for HB recurrence, about 10–20% of HB is associated with lung metastasis, and the overall survival rate of these cases is 25–50% [6, 7]. Hishiki T et al. and Hu HM et al. have reported that 49% and 46.15% of patients with lung metastasis can achieve complete response after chemotherapy without surgery, respectively [7, 8]. Currently, the widely accepted treatment scheme for HB with lung metastasis is chemotherapy and lung tumor resection if lung metastasis remained detectable after chemotherapy [8], followed by complete surgical resection of the liver lesion [9, 10].

Laparoscopy has the benefits of small invasiveness, fast recovery, aesthetic incision, features that make early postoperative chemotherapy possible [11]. Therefore, laparoscopy is increasingly favored by surgeons in pediatric tumor surgery. Although there have been many reports on successful laparoscopy in pediatric neuroblastoma and nephroblastoma [12–14], laparoscopic resection of pediatric HB is still at the exploratory stage and few reports are related to this topic. Smaller liver tumors in the antero-lateral segments (including segments 2, 3, 4b, 5, and 6) are thought to be more easily resected [15]. Kwon H et al. demonstrated laparoscopic liver resection would be a safe and feasible option for liver tumors in children with proper technical efforts and selection of patients [16]. However, whether giant HB in the segments other than antero-lateral segments in children, especially in infants whose weight less than 10 kg, can be treated by laparoscopy remains challenging due to the following reasons: (1) intraoperative hemorrhage may be uncontrollable or even lethal; (2) insufficient residual liver volume leads to postoperative liver failure; (3) whether complete resection and avoidance of recurrence and metastasis are achievable. Infants with HB usually have no cirrhosis and still maintain high liver regeneration ability, and therefore they can tolerate resection of over 60% of the liver volume. Thus, it is believed that if the healthy side of the liver functions well and the patients have no cirrhosis, reducing blood supply to the tumors could raise the safety of the surgery. Breedis C found that 85 to 95% of the blood supply to liver tumors comes from the hepatic arteries [17]. We hypothesized that selective hepatic artery ligation and liver partial partition could reduce the tumor blood supply and further shrinking the tumor volume. In the present study, staged laparoscopic hepatectomy was attempted on three infants with giant HB, and the safety and feasibility of such procedures were discussed.

## Patients and methods

**Fig. 1:**
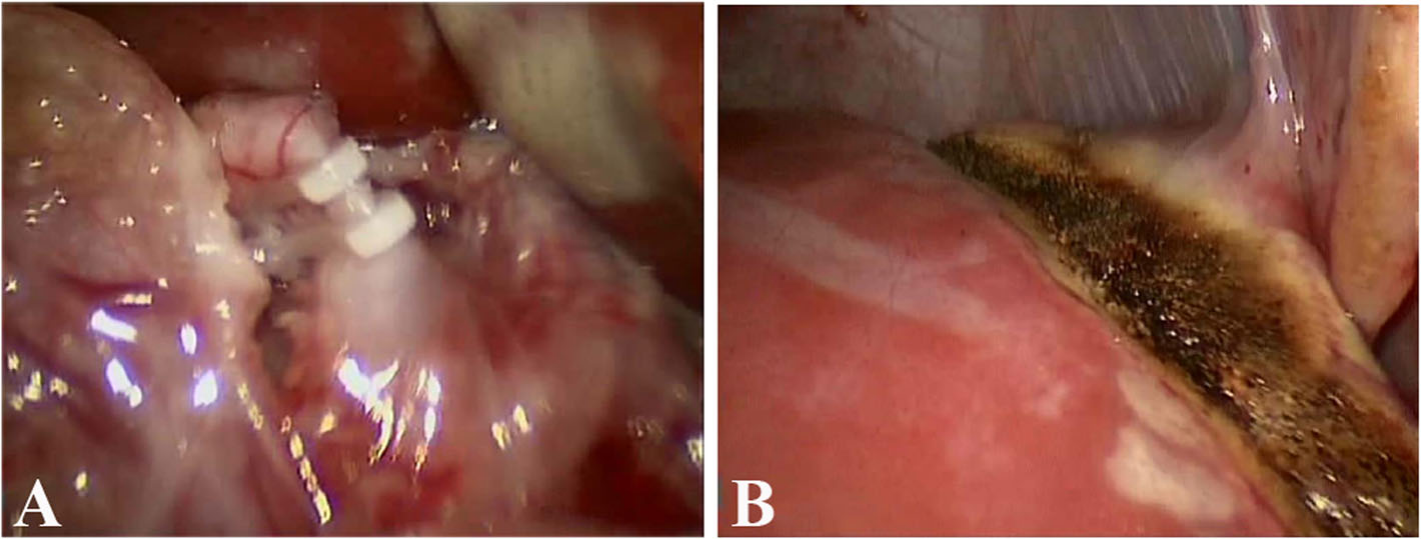
**a** The artery which supplies blood to the tumor (right branch of hepatic artery) was ligated with Hem-O-lok clips under the laparoscope. **b** Liver parenchyma was partially divided with an ultrasonic scalpel, with the dividing depth of about 2.5 cm

From June 2017 to November 2018, 3 infants (1 male and 2 females, aged 6–10 months) with giant HB treated at our department were retrospectively reviewed. They received preoperative chest and abdominal CT scan to evaluate tumor position, size, relationship with surrounding blood vessels, and whether there was distant metastasis. CT scan and Hisense computer assisted surgery (Hisense CAS) system were used to reconstruct the structural relationship between tumor, liver, and blood vessels. All of them were confirmed by B-mode ultrasound-guided biopsy. Formulate chemotherapy according to “2016 Chinese Children Cancer Group Hepatoblastoma Multidisciplinary Diagnosis and Treatment Expert Consensus,” and received 4–5 cycles of neoadjuvant chemotherapy. Stage 1 laparoscopic selective (right or left) hepatic artery ligation and liver partial partition were performed, with the division depth of about 2.5 cm (Fig. 1). The cases were reexamined by abdominal CT at 2 weeks after surgery, and stage 2 laparoscopic (right or left) hemihepatectomy was performed. Four-port laparoscopy was used. In stage 2 surgery, the liver was enveloped by the greater omentum, but the adhesion was easy to dissociate and the original partition line was clearly visible. The Harmonic scalpel (Ethicon Harmonic scalpel™; Ethicon EndoSurgery, USA), monopolar and bipolar electrocautery [18], was used to break the round ligament, the falciform ligament, and the coronary ligament. The right (or left) artery, the right (or left) hepatic duct, and the right branch (or left branch) of the portal vein were separately isolated. When the right liver was resected, several short hepatic veins of caudate lobe were ligated by lifting the liver and separating the space between the inferior vena cava and the right liver. During the dissections, the blood vessels and bile ducts in hepatic parenchyma were ligated one by one with Hem-O-Lock. The right and middle hepatic veins were ligated and the liver was dislocated. When the left hemihepatic liver was performed, the ultrasonic scalpel dissected the liver along the separation zone of stage 1 operation, ligated the perforating vein, bile duct, and the left hepatic vein, removed the left hemihepatic liver at final. The resected specimen was placed into the specimen bag, taken out via an extended incision. The resected tumor was subjected to pathology and classification. The subsequent standardized chemotherapy regimen was determined based on pathology type and staging. After postoperative chemotherapy, the cases received hematologic test and PET/CT scan to evaluate the outcomes. Baseline clinical data, operation time, intraoperative blood loss, intraoperative and postoperative complications, and feeding time after surgery were collected. The cases received follow-up at the outpatient clinic for 18–30 months (average, 24 months).

## Results

The clinical data of the patients is shown in Table 1. There were one male case and two female cases with an average age of 8 months (range, 6–10 months) upon surgery and the average weight was 7.5 kg (range, 5.5–9 kg). Upon the first visit, all cases were estimated of PRETEXT stage III, but one case accompanied by multifocal lung metastases, involvement of the right branch of portal vein and tumor thrombi. After 4–5 cycles of neoadjuvant chemotherapy, all cases were estimated of POSTTEXT stage II. The staged laparoscopic surgery was successful in all three cases. Two cases received right hemihepatectomy and one case left hemihepatectomy, none of which was converted to laparotomy. Postoperative pathology revealed fetal subtype HB in two cases and mixed HB in one case. One week after the stage 1 operation, the levels of alpha-fetoprotein in the three patients were significantly decreased. One week after the complete resection of the tumor in the second-stage operation, the alpha-fetoprotein levels of the three patients were further decreased, and return to normal.

**Table 1:** The clinical data of the patients

Case	Sex	Age at surgery (M)	Weight (kg)	Tumor size at diagnosis (mm)	PRETEXT/POSTEXT staging	Metastatic disease	Chemotherapy	AFP (ng/ml)					Tumor location after chemotherapy	Surgery	Histology
								Initial diagnosis	After chemotherapy	1 week after the first surgery	1 week after the second surgery	Current			
1	Female	10	9	147 × 135 × 105.2	III/ II	M^-^	4 C5VD	15942	1303	987	162	2.12(30 months after surgery)	S2,3,4 of left lobe liver	LH	Embryonal hepatoblastoma
2	Female	6	5.5	85 × 75 × 46	III/II	M^-^	4 C5VD	1054	640	346	157	2.13(18 months after surgery)	S5,8 of right posterior lobe liver	RH	Embryonal hepatoblastoma
3	Male	8	8	154.05 × 98.66 × 86	III/II	M^+^	3 CCD+2 ICE	121000	121000	87560	51623	8.92(24 months after surgery)	right lobe of liver	RH	Mixed-type Hepatoblastoma

*M* Month, *Kg* Kilogram, *MM* Millimeter, *PRETEXT* pre-treatment extent of disease, *POSTEXT* post-treatment extent of disease, *C5VD* Cisplatin, *5-fluorouracil* vincristine and doxorubicin, *ICE* ifosphamide, carboplatin, etoposide, *LH* left hepatectomy, *RH* right hepatectomy, *M*^*+*^ metastatic, *M*^*−*^ no metastatic

**Fig. 2:**
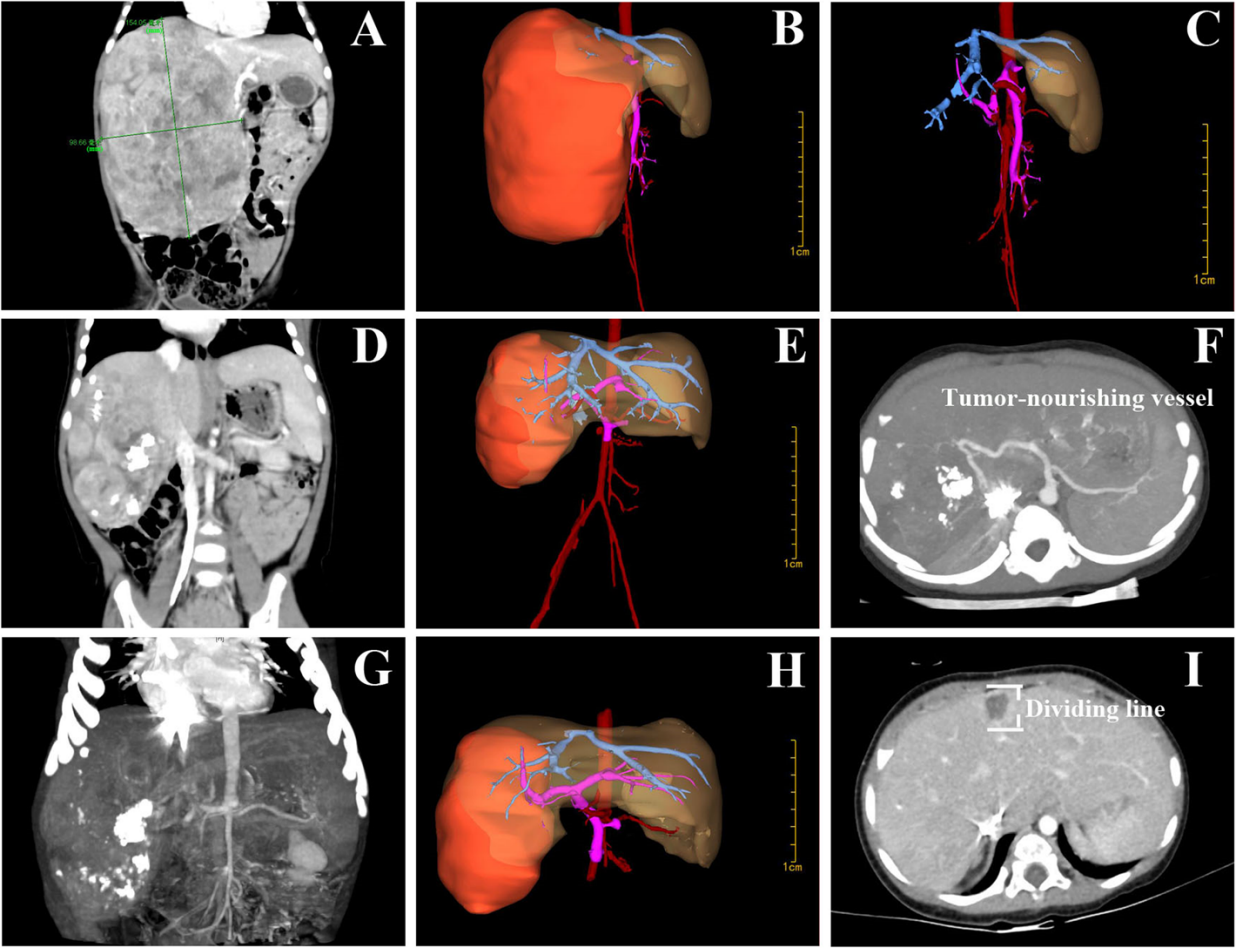
**a** Upon the first visit, CTA revealed giant tumor in the right lobe of liver with unclear boundary, involvement of the right branch of portal vein and tumor thrombi. There were local involvement of the hepatic segment of inferior vena cava and right hepatic vein. There was tumor thrombus in the central vein of liver. **b** The Hisense CAS system reconstructs tumors, liver, and blood vessels. At the time of initial diagnosis, the tumor volume reached 1034.5 ml. **c** The Hisense CAS system simulates hepatectomy, and the residual liver volume percentage is only 18.32%, which cannot meet the needs of liver resection. **d** After neoadjuvant chemotherapy, the tumor volume shrank considerably than before. **e** The Hisense CAS system reconstituted tumors, liver, and blood vessels, and the residual liver volume was increased. **f** CTA revealed that the right branch of hepatic artery supplied blood to the tumor. **g** At 2 weeks after stage 1 laparoscopic right hepatic artery ligation and partial partition of liver parenchyma, the tumor further shrank in size and the intratumoral necrosis further expanded. **h** After reconstruction of the tumor, liver, and blood vessels by the Hisense CAS system, the tumor volume further shrank and the residual liver volume was further increased. The blood flow to the tumor from the right hepatic artery was cut off by the ligation. **i** Postoperative CTA located the partition line in liver parenchyma

The changes in tumor volume at the time of initial diagnosis, after chemotherapy, and after stage 1 operation are shown in Fig. 2 and Table 2. Upon the first visit, the tumor in the liver was giant, vaguely circumscribed and unresectable. At the time of initial diagnosis, the tumor volume of the three patients was 1126.3, 823.25, and 1034.5 ml. The residual liver volume of the three patients was 21%, 25%, and 18.32% by computer-simulated hepatectomy. After 4–5 cycles of neoadjuvant chemotherapy, the tumor shrank in size considerably and its boundary became clearer than before. The residual liver volume of the three children was significantly increased compared with the previous ones by computer-simulated hepatectomy, reaching 36%, 43.3%, and 35%, respectively. After stage 1 laparoscopic selective (right or left) hepatic artery ligation and liver partial partition, the tumor size further reduced while the intratumoral necrosis expanded. The residual liver volume was further increased to 40.02%, 49.14%, and 45% by computer-simulated hepatectomy, respectively.

**Table 2:** Tumor volume measurement

Time	Case 1	Case 2	Case 3
At initial diagnosis (ml)	1126.3	823.25	1034.5
After neoadjuvant chemotherapy (ml)	496.3	342.8	417.2
After stage 1 surgery (ml)	327.8	206.5	302.4

**Fig. 3:**
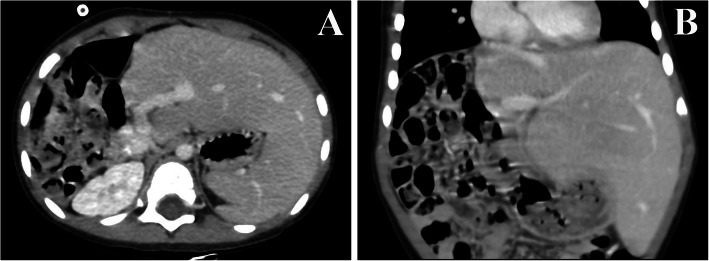
**a** There was no tumor residue or recurrent at 24 months after surgery. **b** The compensatory hypertrophy of the left liver lobe was significant at 24 months after surgery

The operation time of the first surgery was 65–80 min (73 min in average), and the intraoperative blood loss was 15–25 ml (average, 20 ml). The time to start eating was 6 h after surgery in all cases. No biliary fistula or hemorrhage occurred after surgery. The operation time of the stage 2 surgery was 280–335 min (307 min in average), and the intraoperative blood loss was 50–150 ml (average, 95 ml). The time to start eating was 2–3 days (average, 2.67 days) after surgery in all cases. The tumors were resected en bloc without rupture, air embolism, and hemorrhage, neither were there postoperative complications such as infection, biliary fistula, hemorrhage and liver failure. None of the cases had tumor implantation and metastases in the Trocar ports. Twenty-four months after the operation, abdominal CT showed that the residual liver was significantly enlarged, and no tumor recurrence was observed (Fig. 3). The cases were followed up for 18–30 months (average, 24 months). Postoperative chemotherapy was completed. No tumor residue, recurrence, or metastases were found by hematologic test and whole-body PET/CT scan. There was no recurrence or death.

## Discussion

Hepatoblastoma (HB) is the most common liver malignancy in children. Due to hidden symptoms at early stage and inability of infants to speak, many HB lesions are already too large to be resected upon the first visit. Fortunately, HB is usually sensitive to chemotherapy, and the tumor volume may reduce significantly after 3–5 cycles of neoadjuvant chemotherapy, making the lesions fit for resection. In recent years, the rising and popularization of minimally invasive surgical procedure have been witnessed. Although some children with smaller neuroblastoma and nephroblastoma lesions have been successfully treated by laparoscopy, laparoscopic resection of giant HB in infants is still disputable and challenging, with few reports raising the concern of this topic. Moreover, to our knowledge, laparoscopic resection of hepatoblastoma in infants weighing less than 10 kg has been rarely reported previously.

In the present study, staged laparoscopic resection was performed in 3 infants with giant HB. According to the literature, a normal liver can be removed up to 75% of its volume without hepatic insufficiency; but patients with chronic liver disease or chemotherapy should retain > 40% of the liver volume; and for patients with liver cirrhosis, it is often necessary to retain > 50% of the liver volume [18]. It had been shown that 85 to 95% of the blood supply to liver tumors comes from the hepatic arteries [17]. CTA also revealed that the tumors of the three infants were mainly supplied by the hepatic artery; hence, the purpose of stage 1 surgery is to reduce blood supply to the tumors. This will cause the tumor volume to shrink and reduce intraoperative blood loss in stage 2 surgery, thus raising the success rate and lowering the risk of stage 2 resection. Experimental data in mice show ALPPS (Associating Liver Partition and Portal vein ligation for Staged hepatectomy, ALPPS)-induced liver regeneration appears marked increase as compared with portal vein ligation [19]. The underlying mechanisms suggested that circulating inflammatory and growth factors mediate liver regeneration in ALPPS [19]. Specifically, IL-6 and TNF-alpha appeared upregulated after stage 1, with similar observation in humans [19]. Other ALPPS models in rats disclosed analogous results with additionally upregulation of pSTAT3, nuclear NFkBp65, and YAP [20, 21]. Thus, liver partial partition results in the production of circulating inflammatory factors and growth factors that are essential for liver regeneration. Compared with simple selective interventional hepatic artery embolization, selective hepatic artery ligation and liver partial partition can reduce blood supply to the tumor and induce liver regeneration, therefore achieving a better effect. Our clinical results indicated that although the tumor volume shrank significantly after 4–5 cycles of neoadjuvant chemotherapy, the tumors were still too large for one-stage laparoscopic resection. At 2 weeks after laparoscopic selective hepatic artery ligation and liver partial partition, CT scan indicated further tumor shrinkage and the residual liver volume was slightly increased in all three cases. Moreover, the intratumoral necrosis expanded. Hence favorable condition was created for successful stage 2 laparoscopic hemihepatectomy.

If the bile duct is ligated in stage 1 surgery, the risk of biliary fistula, infection, and cholestasis will be increased [22]; if the hepatic artery on the affected side and portal vein are simultaneously ligated, it may cause necrosis of the affected half of the liver and serious consequences. In order to reduce the incidence of complications following the cut of great vessels and bile duct, we only selectively ligated the hepatic artery on the affected side with partial division of liver parenchyma. Meanwhile, fibrin sealant was applied to the wound surface of liver for hemostasis and to prevent adhesion, bleeding, and biliary fistula, and the dividing depth was about 2.5 cm. However, more discussion is needed as to the optimal dividing depth. None of our cases had postoperative complications, such as biliary fistula, bleeding, and infection.

There is worry that the specimen bag may be ruptured, leading to tumor spread and implantation. The conventional method is to make an incision of about 7–8 cm in the lower abdomen to directly take out the entire specimen [23]. During the operation, the resected half of the liver should be carefully placed into the specimen bag and remained intact, and care should be given not to damage the surface of the tumor. As long as the surgical procedures mentioned above are done cautiously, tumor recurrence and implantation at the incision and Trocar ports are very rare [24, 25]. None of the specimen bags ruptured in the present study, and neither were there peritoneal implantation and recurrence at the incision and Trocar ports.

## Conclusion

We reported the resection of giant hepatoblastoma in infants by two-stage laparoscopic hepatectomy associating selective hepatic artery ligation and liver partial partition. This procedure has the benefits of small invasiveness, fast recovery, improved safety and high feasibility. However, the long-term efficacy of this procedure needs to be verified through more cases and longer follow-up. Moreover, suitable cases should be selected in strict accordance with the requirements, and the procedures be performed by experienced surgeons specialized in minimally invasive surgery for pediatric tumors.

## Data Availability

The used data were retrospectively retrieved from electronic medical records of the Sun Yat-Sen Memorial Hospital of Sun Yat-Sen University under the requests and approval of IRB. Further, it was claimed that the data that support the findings of this study can only be accessed by the researchers and assistants in the team. Feel free to contact the corresponding authors regarding the availability of data and materials.
